# The morbidity and mortality of COVID-19 are correlated with the Ile105Val glutathione *S*-transferase P1 polymorphism

**DOI:** 10.1186/s43042-020-00094-0

**Published:** 2020-10-01

**Authors:** Mostafa Saadat

**Affiliations:** Department of Biology, College of Sciences, Shiraz University, Shiraz, 71467-13565 Iran

**Keywords:** Ecologic study, Epidemiologic measures, Pandemic

## Abstract

**Background:**

Oxidative stress is an important issue in coronavirus disease 2019 (COVID-19). Considering that glutathione *S*-transferase P1 (*GSTP1*) is involved in cellular detoxification, it may play an important role in susceptibility to infection with SARS-CoV-2 and/or its outcome. In the present study, the association between the Ile105Val *GSTP1* polymorphism (rs1695) and susceptibility to SARS-CoV-2 infection, as well as its outcome was investigated. Data on the prevalence (per 10^6^ people), case-fatality (per 100 infected cases), and mortality (per 10^6^ people) of COVID-19 and various potential confounders (the life expectancy at birth, density of medical doctors, density of nursing and midwifery personnel, and the gross national income per capita) were used. The latest data available for 45 countries were used for the study.

**Results:**

In multivariate linear regression analyses, the Val105 allelic frequency showed positive association with the log-prevalence (partial *r* = 0.308, *p* = 0.042) and log-mortality of COVID-19 (partial *r* = 0.316, *p* = 0.037). The log-fatality did not show association with the allelic frequency. In the next step, only countries with the gross national income per capita more than $15,000 were included in the analysis. In the selected countries, the frequency of Val105 was positively associated with the log-prevalence (partial *r* = 0.456, *p* = 0.009) and log-mortality of COVID-19 (partial *r* = 0.544, *p* = 0.001).

**Conclusions:**

The present findings indicate that countries with higher Val105 allelic frequency of the rs1695 polymorphism showed higher prevalence and mortality of COVID-19.

## Background

Numerous gene families, including glutathione *S*-transferases (GSTs) superfamily, are involved in cellular detoxification process and neutralizing oxidative stress. The *GSTP1* (MIM: 134660, belong to class pi) has many genetic variations in human. A missense variant A/G (rs1695) in exon 5 of the *GSTP1* result in amino acid substitution Ile105Val. The stability of the Val105 form is less than the other form [1]. This alteration decreased the GSTP1 enzyme activity [2–4].

The GSTP1 is expressed in all tissues and cells, including lung epithelial cells and lung-resident macrophages [5–7]. In occupationally di-isocyanate-induced asthma, the Val105 allele is positively associated with the bronchial hyperreactivity [8]. Exposure to high “diesel exhaust particle” increased the risk of wheezing phenotypes only among the carriers of the Val105 allele (Ile/Val and Val/Val genotypes) [9]. Children with the Val/Val genotype had slower lung function growth than the other genotypes [10]. Downregulation of the *GSTP1* mRNA level and lower total GST activity were reported in a mouse model of asthma following allergen challenge [11]. Taken together, it is concluded that the GSTP1 has a central role in lung function.

Coronavirus disease 2019 (COVID-19) is a contagious disease; therefore, its prevalence (per 10^6^ people), case-fatality (per 100 infected cases), and mortality (per 10^6^ people) may be affected by various social factors, such as the life expectancy at birth, country income, etc. Very recently some investigators suggested that some genetic polymorphisms might be involved in susceptibility to genetic polymorphisms or outcome of the disease [12–16].

Oxidative stress is an important issue in COVID-19 [17–19]. Considering that GSTP1 is involved in cellular detoxification and it has important role in lung function, it is suggested that GSTP1 plays an important role in susceptibility to SARS-CoV-2 infection and/or its outcome. Very recently, association between some genetic polymorphisms and morbidity/mortality of COVID-19 were reported [12–16]. A study showed that the COVID-19 mortality and case-fatality are associated with the *GSTT1* polymorphism [12]. There are no data on the association between COVID-19 epidemiologic parameters and the rs1695. These facts sufficiently provide us with a theoretical hypothesis to carry out the present study.

## Methods

In this study, the life expectancy at birth (LE), density of medical doctors, density of nursing and midwifery personnel, and the gross national income (GNI) per capita (PPP international $) as the indices for economic situation and health services in different countries were considered as the potential risk factors for susceptibility to COVID-19 or the disease outcome. The latest data available for countries were achieved from the World Health Organization website www.who.int/countries/en/. The latest data for GNI per capita for countries were obtained from the World Bank data (https://data.worldbank.org/indicator/NY.GNP.PCAP.PP.CD).

The number of COVID-19 diagnostic tests performed per one million population in each country was also used as another risk variable. Data for the prevalence, fatality, mortality, and level of performed diagnostic test of COVID-19 on July 7, 2020, were achieved from the website www.worldo meters.info/coronavirus/countries. The Val105 allelic frequency in different countries was obtained from previous reports (Table S1 in supplement file). Data from 45 countries were included in the analysis. Data from Argentina, Australia, Brazil, Bulgaria, Canada, China, Colombia, Czech, Denmark, Egypt, Finland, France, Germany, Hungary, Iceland, India, Iran, Iraq, Italy, Jamaica, Japan, Jordan, Kazakhstan, Lebanon, Mexico, Moldova, Morocco, Netherland, Norway, Poland, Portugal, Romania, Russia, Saudi Arabia, Serbia, Singapore, Slovenia, South Africa, South Korea, Spain, Sweden, Thailand, Turkey, the UK, and the USA were included in the analysis.

Variables were checked for their normality by one-sample Kolmogorov-Smirnov test. Non-normally distributed variables (prevalence, mortality, case-fatality rates, and number diagnostic test performed per 10^6^ people) were log-transformed.

Variables with *p* ≤ 0.1 in the univariable analysis were introduced into the multivariable models. Only two different models were fitted for combination of the epidemiologic parameters with the rs1695 polymorphism. The log-transformed variables were considered as outcome variables, and the allelic frequency and the risk factors were introduced into the model as explanatory variables. A backward removal method was used for each model construction. Analyses were performed using the SPSS statistical soft-ware (Chicago, IL, USA, version 24). A *p* < 0.05 was considered statistically significant difference

## Results

In univariate analysis, the frequency of the Val105 allele showed no significant correlations with the log-prevalence (*r* = 0.331, df = 44, *p* = 0.025), log-mortality (*r* = 0.363, df = 44, *p* = 0.013), and log-fatality (*r* = 0.175, df = 44, *p* = 0.244) of the COVID-19.

Table 1 shows the association between potential risk factors and the log-transformed of prevalence, mortality, and case-fatality of COVID-19. Variables with *p* ≤ 0.1 in the univariable analysis were used in multivariable linear regression analyses (Table 1).

**Table 1:** Correlation between log-transformed of epidemiologic parameters and selected risk factors

Variables	Log-Prevalence		Log-Mortality		Log-Fatality	
	*r*	*P*	*r*	*P*	*r*	*p*
Log-GNI per capita	0.406	0.006	0.349	0.019	0.065	0.673
Log-number of COVID-19 diagnostic tests performed (per 10^6^ people)	0.443	0.002	0.241	0.111	− 0.159	0.297
Life expectancy at birth (years)	0.091	0.551	0.187	0.220	0.193	0.204
Density of medical doctors (per 10^4^ people)	0.384	0.009	0.393	0.008	0.162	0.287
Density of nursing and midwifery personnel (per 10^4^ people)	0.288	0.055	0.251	0.096	0.048	0.752

*Degree of freedom (df) for all correlations is 43

Two different models were fitted for combination of prevalence and mortality with the rs1695 polymorphism. The log-prevalence and log-mortality were considered as outcome variables, and the Val105 allelic frequency and the risk factors were introduced into the model as explanatory variables.

Results of multivariate analysis are summarized in Table 2. Based on multivariate analyses, the frequency of Val105 was positively associated with the log-mortality (partial *r* = 0.316, *p* = 0.037) and log-prevalence of COVID-19 (partial *r* = 0.308, *p* = 0.042).

**Table 2:** Multivariable linear regression analysis for associations of log-mortality and log-prevalence of COVID-19 with the frequency of the Val105 allele in the various countries around the world

Variables	Unstandardized coefficients		Standardized coefficients beta	Partial correlations	*t*	*P*
	*B*	Std. Error				
Log-prevalence as dependent variable						
Constant	− 1.067	1.231	-	-	− 0.867	0.391
Log-GNI per capita	0.815	0.262	0.407	0.424	3.034	0.004
Val105 allelic frequency	0.020	0.009	0.281	0.308	2.094	0.042
Log-mortality as dependent variable						
Constant	− 3.2348	1.648	-	-	− 1.971	0.055
Log-GNI per capita	0.918	0.359	0.350	0.367	2.553	0.014
Val105 allelic frequency	0.027	0.013	0.296	0.316	2.156	0.037

The first model was significant with *F* = 6.76; df = 2, 42; *P* = 0.003; adjusted *R*^2^ = 0.207. The second model was significant with *F* = 5.55; df = 2, 42; *P* = 0.007; adjusted *R*^2^ = 0.171

In next step, only countries with the gross national income (GNI) per capita (PPP international $) more than $15,000 were included in the analysis. Table 3 summarizes the associations between the risk factors and the log-transformed of epidemiologic parameters of COVID-19 in the selected countries. The results of multivariate analysis are summarized in Table 4. In the selected countries, the frequency of Val105 was positively associated with the log-prevalence (partial *r* = 0.456, *p* = 0.009) and log-mortality of COVID-19 (partial *r* = 0.544, *p* = 0.001).

**Table 3:** Correlation between log-transformed of epidemiologic parameters and possible confounders in selected countries with GNI-per capita more than $15,000

Variables	Log-Prevalence		Log-Mortality		Log-Fatality	
	*r*	*P*	*r*	*P*	*r*	*p*
Log-GNI per capita	0.527	0.001	0.353	0.041	− 0.084	0.637
Log-number of COVID-19 diagnostic tests performed (per 10^6^ people)	0.541	0.001	0.261	0.136	− 0.230	0.190
Life expectancy at birth (years)	0.141	0.426	0.240	0.171	0.191	0.278
Density of medical doctors (per 10^4^ people)	0.469	0.005	0.461	0.006	0.139	0.434
Density of nursing and midwifery personnel (per 10^4^ people)	0.243	0.166	0.172	0.332	− 0.030	0.866

Degree of freedom (df) for all correlations is 32

**Table 4:** Multivariable linear regression analysis for associations of log-mortality and log-prevalence of COVID-19 with the frequency of the Val105 allele in the various countries around the world in selected countries with GNI-per capita more than $15,000

Variables	Unstandardized coefficients		Standardized coefficients beta	Partial correlations	*t*	*P*
	*B*	Std. Error				
Log-prevalence as dependent variable						
Constant	− 4.790	1.717	-	-	− 2.789	0.009
Log-test	0.429	0.194	0.319	0.374	2.209	0.035
Log-GNI per capita	1.118	0.417	0.384	0.440	2.681	0.012
Val105 allelic frequency	0.028	0.010	0.360	0.456	2.806	0.009
Log-mortality as dependent variable						
Constant	− 5.979	2.463	-	-	− 2.427	0.021
Log-GNI per capita	1.344	0.530	0.358	0.415	2.537	0.016
Val105 allelic frequency	0.051	0.014	0.509	0.544	3.606	0.001

The first model was significant with *F* = 10.726; df = 3, 30; *P* < 0.001; adjusted *R*^2^ = 0.469. The second model was significant with *F* = 9.63; df = 2, 31; *P* < 0.001; adjusted *R*^2^ = 0.344

## Discussion

The main findings of the present study are that the frequency of Val105 was positively associated with the log-prevalence and log-mortality of COVID-19. It means that countries with higher Val105 allelic frequency showed higher prevalence of COVID-19 and mortality due to COVID-19. The present findings may explain, at least in part, some differences in COVID-19 mortality between East Asian and European populations by the Val105 allelic frequency.

Numerous facts are indicated that GSTP1 plays an important role in lung function [5, 8–11, 20]. GSTP1 has pleiotropic properties; it binds directly to c-Jun N-terminal kinases and acts as a negative regulator. It also has a negative regulatory role in regulating tumor necrosis factor-alpha (TNFα)-induced MAPK signaling. These functions are independent from its enzyme activity [21].

It is widely acknowledged that the mortality due to severe respiratory problems in patients infected by SARS-CoV-2 is high [22]. On the other hand, COVID-19 is associated with oxidative stress [17, 18]. It is suggested that COVID-19 mortality might be associated with oxidative stress and/or SARS-CoV-2-activated cytokine storm syndrome. Both of these phenomena might be interpreted with the pleiotropy properties of *GSTP1*.

It is worth mentioning that the Val105 form compared to the other form has lower detoxification activity; therefore, COVID-19 patients, who are carriers of Val105, often experience severe oxidative stress compared to the Ile/Ile genotype. This may lead to severity of the disease and subsequently death due to COVID-19 in the carriers of Val105.

A literature review indicated that COVID-19 has affected more males than females, and also male gender significantly increases the case fatality [23, 24]. This difference, at least in part, may be explained by the sexual dimorphisms of GSTP1 enzyme activity. The enzyme activity is higher in females than males [25]. Several observational and experimental studies should be carried out to approve the present findings and the abovementioned hypotheses.

## Conclusions

The present findings indicate that countries with Val105 higher allelic frequency of the rs1695 polymorphism showed higher prevalence and mortality of COVID-19.

## Supplementary information

**Additional file 1: Table S1.** Prevalence, mortality and case-fatality of COVID-19 in 46 countries, the genotypic frequency of the *GSTP1* Ile105Val polymorphism and other variables used in the study

## Data Availability

All data generated or analyzed during this study are included in this published article and its supplementary information file.

## Abbreviations

COVID-19: Coronavirus disease 2019
df: Degree of freedom
*GSTP1*: Glutathione *S*-transferase P1
GNI: Gross national income

## References

[1] Johansson AS, Stenberg G, Widersten M, Mannervik B (1998) Structure-activity relationships and thermal stability of human glutathione transferase P1-1 governed by the H-site residue 105. J Mol Biol 278:687–698 9600848 10.1006/jmbi.1998.1708

[2] Ali-Osman F, Akande O, Antoun G, Mao JX, Buolamwini J (1997) Molecular cloning, character-rization, and expression in Escherichia coli of full-length cDNAs of three human glutathione S-transferase Pi gene variants. Evidence for differential catalytic activity of the encoded proteins. J Biol Chem 272:10004–10012 9092542 10.1074/jbc.272.15.10004

[3] Sundberg K, Johansson AS, Stenberg G et al (1998) Differences in the catalytic efficiencies of allelic variants of glutathione transferase P1-1 towards carcinogenic diol epoxides of polycyclic aromatic hydrocarbons. Carcinogenesis 19:433–436 9525277 10.1093/carcin/19.3.433

[4] Zimniak P, Nanduri B, Pikula S et al (1994) Naturally occurring human glutathione S-transferase GSTP1-1 isoforms with isoleucine and valine in position 104 differ in enzymic properties. Eur J Biochem 224:893–899 7925413 10.1111/j.1432-1033.1994.00893.x

[5] Terrier P, Townsend AJ, Coindre JM et al (1990) An immunohistochemical study of pi class glutathione S-transferase expression in normal human tissue. Am J Pathol 137:845–853 1977319 PMC1877535

[6] Moscow JA, Fairchild CR, Madden MJ et al (1989) Expression of anionic glutathione-S-transferase and P-glycoprotein genes in human tissues and tumors. Cancer Res 49:1422–1428 2466554

[7] Saint-Georges F, Abbas I, Billet S et al (2008) Gene expression induction of volatile organic compound and/or polycyclic aromatic hydrocarbon-metabolizing enzymes in isolated human alveolar macrophages in response to airborne particulate matter (PM2.5). Toxicology 244:220–230 18178302 10.1016/j.tox.2007.11.016

[8] Leppilahti J, Majuri ML, Sorsa T et al (2019) Associations between glutathione-S-transferase genotypes and bronchial hyperreactivity patients with di-isocyanate induced asthma. A follow-up study. Front Med (Lausanne) 6:220 31649932 10.3389/fmed.2019.00220 PMC6794415

[9] Schroer KT, Biagini Myers JM, Ryan PH et al (2009) Associations between multiple environmental exposures and glutathione S-transferase P1 on persistent wheezing in a birth cohort. J Pediatr 154:401–408.e1 18950799 10.1016/j.jpeds.2008.08.040 PMC2783998

[10] Gilliland FD, Gauderman WJ, Vora H, Rappaport E, Dubeau L (2002) Effects of glutathione S-transferase M1, T1, and P1 on childhood lung function growth. Am J Respir Crit Care Med 166:710–716 12204870 10.1164/rccm.2112065

[11] Schroer KT, Gibson AM, Sivaprasad U et al (2011) Downregulation of glutathione S-transferase pi in asthma contributes to enhanced oxidative stress. J Allergy Clin Immunol 128:539–548 21570714 10.1016/j.jaci.2011.04.018 PMC3164907

[12] Saadat M (2020) An evidence for correlation between the glutathione S-transferase T1 (GSTT1) polymorphism and outcome of COVID-19. Clin Chim Acta 508:213–216 32454047 10.1016/j.cca.2020.05.041 PMC7245315

[13] Saadat M (2020) No significant correlation between ACE Ins/Del genetic polymorphism and COVID-19 infection. Clin Chem Lab Med 58:1127–1128 32386188 10.1515/cclm-2020-0577

[14] Ansari-Lari M, Saadat M (2020) The morbidity and mortality of COVID-19 are associated with ABO and Rh blood groups. Eur J Prev Cardiol 7:2047487320939216. 10.1177/2047487320939216 10.1177/2047487320939216 PMC8500000 34551081

[15] Kuo CL, Pilling LC, Atkins JL, et al. APOE e4 genotype predicts severe COVID-19 in the UK Biobank community cohort. J Gerontol A Biol Sci Med Sci 2020; glaa131, Doi: 10.1093/gerona/glaa131 10.1093/gerona/glaa131 PMC7314139 32451547

[16] Delanghe JR, De Buyzere ML, Speeckaert MM (2020) C3 and ACE1 polymorphisms are more important confounders in the spread and outcome of COVID-19 in comparison with ABO polymorphism. Eur J Prev Cardiol 27:1331–1332 32460534 10.1177/2047487320931305 PMC7717311

[17] Khomich O, Kochetkov S, Bartosch B, Ivanov AV (2018) Redox biology of respiratory viral infections. Viruses 10:392 30049972 10.3390/v10080392 PMC6115776

[18] Henry BM, Vikse J, Benoit S et al (2020) Hyperinflammation and derangement of renin-angiotensin-aldosterone system in COVID-19: A novel hypothesis for clinically suspected hypercoagulopathy and micro-vascular immunothrombosis. Clin Chim Acta 507:167–173 32348783 10.1016/j.cca.2020.04.027 PMC7195008

[19] Shneider A, Kudriavtsev A, Vakhrusheva A (2020) Can melatonin reduce the severity of COVID-19 pandemic? Int Rev Immunol 29:1–10 10.1080/08830185.2020.1756284 32347747

[20] Piacentini S, Polimanti R, Simonelli I et al (2013) Glutathione S-transferase polymorphisms, asthma susceptibility and confounding variables: a meta-analysis. Mol Biol Rep 40:3299–3313 23307299 10.1007/s11033-012-2405-2

[21] Zhang J, Grek C, Ye ZW et al (2014) Pleiotropic functions of glutathione S-transferase P. Adv Cancer Res 122:143–175 24974181 10.1016/B978-0-12-420117-0.00004-9 PMC5079281

[22] de Lusignan S, Dorward J, Correa A, et al. Risk factors for SARS-CoV-2 among patients in the Oxford Royal College of general practitioners research and surveillance center primary care network: a cross-sectional study. Lancet Infect Dis 2020;S1473-3099(20)30371-6. 10.1016/S1473-3099(20)30371-6 PMC7228715 32422204

[23] Kowalewski M, Fina D, Słomka A et al (2020) COVID-19 and ECMO: the interplay between coagulation and inflammation-a narrative review. Crit Care 24:205 32384917 10.1186/s13054-020-02925-3 PMC7209766

[24] Nikpouraghdam M, Jalali Farahani A, Alishiri G et al (2020) Epidemiological characteristics of coronavirus disease 2019 (COVID 19) patients in IRAN: a single center study. J Clin Virol 127:104378 32353762 10.1016/j.jcv.2020.104378 PMC7172806

[25] Wang L, Ahn YJ, Asmis R (2020) Sexual dimorphism in glutathione metabolism and glutathione-dependent responses. Redox Biol 31:10410 10.1016/j.redox.2019.101410 PMC7212491 31883838

