# Supplementary material for: The morbidity and mortality of COVID-19 are correlated with the Ile105Val glutathione S-transferase P1 polymorphism
Source: Egypt J Med Hum Genet. 2020 Oct 1;21(1):52. doi: 10.1186/s43042-020-00094-0 (PMC7527247; doi:10.1186/s43042-020-00094-0)
Supplement: Supplementary file 1 — Additional file 1: Table S1. Prevalence, mortality and case-fatality of COVID-19 in 46 countries, the genotypic frequency of the GSTP1 Ile105Val polymorphism and other variables used in the study [file 43042_2020_94_MOESM1_ESM.doc]

**Table S1:** Prevalence, mortality and case-fatality of COVID-19 in 46 countries, the genotypic frequency of the *GSTP1* Ile105Val polymorphism and other variables used in the study

| Country | Prevalence  (per 10^6^ population) | Mortality (per 10^6^ population) | Case-fatality (per 100 infected cases) | Number of COVID-19 diagnostic tests performed (per 10^6^ population) | *GSTP1* Val105 allele (%) | Life expectancy at birth (Years) | Gross national income per capita (PPP international $) | Density of medical doctors (per 10^4^ population) | Density of nursing and midwifery personnel (per 10^4^ population) |
| --- | --- | --- | --- | --- | --- | --- | --- | --- | --- |
| Argentina | 1846 | 36 | 1.97 | 9174 | 32.51 | 76.9 | 22470 | 39.6 | 25.8 |
| Australia | 343 | 4 | 1.21 | 109785 | 34.55 | 82.9 | 49440 | 35.9 | 126.6 |
| Brazil | 7878 | 315 | 3.99 | 20304 | 31.30 | 75.1 | 14530 | 21.5 | 97.1 |
| Bulragia | 878 | 37 | 4.16 | 23005 | 26.59 | 74.9 | 22650 | 39.9 | 53.0 |
| Canada | 2813 | 231 | 8.20 | 80009 | 35.58 | 82.8 | 49430 | 26.1 | 99.1 |
| China | 58 | 3 | 5.55 | 62814 | 18.45 | 76.4 | 15320 | 17.9 | 23.1 |
| Colombia | 2446 | 86 | 3.50 | 17730 | 37.62 | 75.1 | 14310 | 20.8 | 12.6 |
| Czechia | 1184 | 33 | 2.77 | 53670 | 31.72 | 79.1 | 38180 | 43.1 | 84.1 |
| Denmark | 2225 | 105 | 4.73 | 199841 | 33.39 | 81.2 | 57980 | 44.6 | 103.0 |
| Egypt | 755 | 34 | 4.51 | 1319 | 13.67 | 70.5 | 11350 | 7.9 | 14.0 |
| Finland | 1311 | 59 | 4.53 | 46112 | 25.88 | 81.4 | 49720 | 38.1 | 147.2 |
| France | 2586 | 459 | 17.73 | 21212 | 31.38 | 82.9 | 47490 | 32.3 | 96.9 |
| Germany | 2367 | 109 | 4.59 | 70099 | 31.92 | 80.9 | 55980 | 42.1 | 132.0 |
| Hungary | 435 | 61 | 14.01 | 29709 | 32.51 | 76.0 | 30310 | 32.3 | 66.4 |
| Iceland | 5488 | 29 | .53 | 267666 | 33.42 | 82.4 | 55920 | 39.7 | 156.8 |
| India | 539 | 15 | 2.78 | 7398 | 26.61 | 68.8 | 6630 | 7.8 | 21.1 |
| Iran | 2925 | 142 | 4.86 | 21983 | 26.88 | 75.7 | 14560 | 11.4 | 18.7 |
| Iraq | 1608 | 67 | 4.15 | 15521 | 18.82 | 69.9 | 10780 | 8.2 | 16.8 |
| Italy | 4002 | 577 | 14.42 | 94338 | 35.01 | 82.7 | 43260 | 40.9 | 58.7 |
| Jamaica | 249 | 3 | 1.36 | 9049 | 49.53 | 76.0 | 9520 | 13.2 | 11.4 |
| Japan | 158 | 8 | 4.89 | 4033 | 14.25 | 84.2 | 43010 | 24.1 | 115.2 |
| Jordan | 115 | 1 | .86 | 42558 | 27.09 | 74.3 | 10050 | 23.4 | 33.9 |
| Kazakhstan | 2645 | 14 | .53 | 86274 | 18.61 | 71.1 | 22950 | 32.5 | 84.9 |
| Lebanon | 279 | 5 | 1.89 | 22321 | 30.10 | 76.3 | 16330 | 22.7 | 26.4 |
| Mexico | 2030 | 241 | 11.89 | 4972 | 51.58 | 76.6 | 19870 | 22.5 | 29.0 |
| Moldova | 4497 | 149 | 3.32 | 25083 | 30.37 | 71.5 | 13170 | 32.0 | 45.1 |
| Morocco | 396 | 7 | 1.64 | 21705 | 32.86 | 76.0 | 8430 | 7.3 | 11.0 |
| Netherland | 2958 | 358 | 12.10 | 35970 | 32.68 | 81.6 | 58140 | 35.1 | 111.0 |
| Norway | 1650 | 46 | 2.81 | 66060 | 34.66 | 82.5 | 70530 | 46.3 | 181.2 |
| Poland | 962 | 40 | 4.20 | 44295 | 32.26 | 77.7 | 30410 | 24.0 | 57.2 |
| Portugal | 4356 | 160 | 3.67 | 124698 | 33.27 | 81.4 | 33520 | 33.4 | 63.7 |
| Romania | 1540 | 94 | 6.07 | 40726 | 19.15 | 75.2 | 28350 | 22.6 | 61.0 |
| Russia | 4757 | 72 | 1.51 | 147584 | 45.64 | 72.0 | 27840 | 40.1 | 86.2 |
| Saudi Arabia | 6235 | 58 | .93 | 57972 | 30.64 | 74.8 | 49200 | 23.9 | 57.0 |
| Serbia | 1914 | 38 | 1.97 | 52149 | 34.01 | 76.3 | 16710 | 31.3 | 61.2 |
| Singapore | 7715 | 4 | .06 | 129503 | 19.07 | 82.9 | 92150 | 23.1 | 72.1 |
| Slovenia | 836 | 53 | 6.38 | 52717 | 33.02 | 80.9 | 38140 | 30.0 | 96.8 |
| South Africa | 3639 | 59 | 1.62 | 32156 | 45.01 | 63.6 | 12530 | 9.1 | 35.2 |
| South Korea | 257 | 6 | 2.16 | 26257 | 19.23 | 82.7 | 39630 | 23.7 | 69.7 |
| Spain | 6400 | 607 | 9.49 | 122652 | 31.58 | 83.0 | 40570 | 40.7 | 55.3 |
| Sweden | 7261 | 539 | 7.43 | 51503 | 30.55 | 82.3 | 54640 | 54.0 | 115.4 |
| Thailand | 46 | 1 | 1.82 | 8648 | 27.31 | 75.5 | 17650 | 8.1 | 29.6 |
| Turkey | 2465 | 62 | 2.53 | 44257 | 34.92 | 76.4 | 27710 | 17.6 | 26.3 |
| UK | 4218 | 654 | 15.50 | 158741 | 34.44 | 81.4 | 46240 | 28.1 | 82.9 |
| USA | 9356 | 405 | 4.33 | 117211 | 34.13 | 78.6 | 63780 | 25.9 | 85.5 |

**References**

Agúndez JA, García-Martín E, Martínez C, et al. The GSTP1 gene variant rs1695 is not associated with an increased risk of multiple sclerosis. *Cell Mol Immunol*. 2015;12(6):777‐779. doi:10.1038/cmi.2014.121

Al-Eitan LN, Rababa'h DM, Alghamdi MA, Khasawneh RH. Association Of GSTM1, GSTT1 And GSTP1 Polymorphisms With Breast Cancer Among Jordanian Women. *Onco Targets Ther*. 2019;12:7757‐7765. Published 2019 Sep 20. doi:10.2147/OTT.S207255

Coral-Vázquez RM, Romero Arauz JF, Canizales-Quinteros S, et al. Analysis of polymorphisms and haplotypes in genes associated with vascular tone, hypertension and oxidative stress in Mexican-Mestizo women with severe preeclampsia. *Clin Biochem*. 2013;46(7-8):627‐632. doi:10.1016/j.clinbiochem.2012.12.016

Galván CA, Elbarcha OC, Fernández EJ, Beltramo DM, Soria NW. Genetic profiling of GSTP1, DPYD, FCGR2A, FCGR3A and CCND1 genes in an Argentinian population. *Clin Biochem*. 2011;44(13):1058‐1061. doi:10.1016/j.clinbiochem.2011.06.080

Han LY, Liu K, Lin XL, Zou BB, Zhao JS. Lack of any association of GST genetic polymorphisms with susceptibility to ovarian cancer--a meta-analysis. *Asian Pac J Cancer Prev*. 2014;15(15):6131‐6136. doi:10.7314/apjcp.2014.15.15.6131

Iskakova AN, Romanova AA, Aitkulova AM, Sikhayeva NS, Zholdybayeva EV, Ramanculov EM. Polymorphisms in genes involved in the absorption, distribution, metabolism, and excretion of drugs in the Kazakhs of Kazakhstan. *BMC Genet*. 2016;17:23. Published 2016 Jan 19. doi:10.1186/s12863-016-0329-x

Kiyohara C, Horiuchi T, Takayama K, Nakanishi Y. Genetic polymorphisms involved in carcinogen metabolism and DNA repair and lung cancer risk in a Japanese population. *J Thorac Oncol*. 2012;7(6):954‐962. doi:10.1097/JTO.0b013e31824de30f

Kuang M, Xu W, Cao CX, et al. Glutathione S-transferase P1 rs1695 A>G polymorphism and breast cancer risk: evidence from a meta-analysis. *Genet Mol Res*. 2016;15(2):10.4238/gmr.15027771. Published 2016 Jun 16. doi:10.4238/gmr.15027771

Lautner-Csorba O, Gézsi A, Erdélyi DJ, et al. Roles of genetic polymorphisms in the folate pathway in childhood acute lymphoblastic leukemia evaluated by Bayesian relevance and effect size analysis. *PLoS One*. 2013;8(8):e69843. Published 2013 Aug 5. doi:10.1371/journal.pone.0069843

MacIntyre EA, Brauer M, Melén E, et al. GSTP1 and TNF Gene variants and associations between air pollution and incident childhood asthma: the traffic, asthma and genetics (TAG) study. *Environ Health Perspect*. 2014;122(4):418‐424. doi:10.1289/ehp.1307459

Mandal RK, Mittal RD. Glutathione S-Transferase P1 313 (A > G) Ile105Val Polymorphism Contributes to Cancer Susceptibility in Indian Population: A Meta-analysis of 39 Case-Control Studies. *Indian J Clin Biochem*. 2020;35(1):8‐19. doi:10.1007/s12291-018-0787-1

Martínez-Ramírez OC, Pérez-Morales R, Castro C, et al. Polymorphisms of catechol estrogens metabolism pathway genes and breast cancer risk in Mexican women. *Breast*. 2013;22(3):335‐343. doi:10.1016/j.breast.2012.08.004

Massabayeva M, Chaizhunusova N, Aukenov N, et al. Association of radiation risk in the second and third generations with polymorphisms in the genes CYP1A1, CYP2E1, GSTP1 and changes in the thyroid. *Mol Med*. 2019;25(1):48. Published 2019 Nov 14. doi:10.1186/s10020-019-0117-y

Medhasi S, Pasomsub E, Vanwong N, et al. Clinically relevant genetic variants of drug-metabolizing enzyme and transporter genes detected in Thai children and adolescents with autism spectrum disorder. *Neuropsychiatr Dis Treat*. 2016;12:843‐851. Published 2016 Apr 13. doi:10.2147/NDT.S101580

Reszka E, Jablonowski Z, Wieczorek E, et al. Polymorphisms of NRF2 and NRF2 target genes in urinary bladder cancer patients. *J Cancer Res Clin Oncol*. 2014;140(10):1723‐1731. doi:10.1007/s00432-014-1733-0

Rezaei M, Saadat M. Association Between GSTP1 Ile105Val Genetic Polymorphism and Dependency to Heroin and Opium. *Biochem Genet*. 2019;57(2):214‐221. doi:10.1007/s10528-018-9885-2

Ross-Hansen K, Linneberg A, Johansen JD, et al. The role of glutathione S-transferase and claudin-1 gene polymorphisms in contact sensitization: a cross-sectional study. *Br J Dermatol*. 2013;168(4):762‐770. doi:10.1111/bjd.12126

Saadat M. Evaluation of glutathione S-transferase P1 (GSTP1) Ile105Val polymorphism and susceptibility to type 2 diabetes mellitus, a meta-analysis. *EXCLI J*. 2017;16:1188‐1197. Published 2017 Nov 6. doi:10.17179/excli2017-828

Song QB, Wang Q, Hu WG. A systemic review of glutathione S-transferase P1 Ile105Val polymorphism and colorectal cancer risk. *Chin J Cancer Res*. 2014;26(3):255‐267. doi:10.3978/j.issn.1000-9604.2014.06.01

Song Z, Shao C, Feng C, Lu Y, Gao Y, Dong C. Association of glutathione S-transferase T1, M1, and P1 polymorphisms in the breast cancer risk: a meta-analysis. *Ther Clin Risk Manag*. 2016;12:763‐769. Published 2016 May 12. doi:10.2147/TCRM.S104339

Tecza K, Pamula-Pilat J, Kolosza Z, Radlak N, Grzybowska E. Genetic polymorphisms and gene-dosage effect in ovarian cancer risk and response to paclitaxel/cisplatin chemotherapy. *J Exp Clin Cancer Res*. 2015;34(1):2. Published 2015 Jan 16. doi:10.1186/s13046-015-0124-y

Varzari A, Deyneko IV, Tudor E, Turcan S. Polymorphisms of glutathione S-transferase and methylenetetrahydrofolate reductase genes in Moldavian patients with ulcerative colitis: Genotype-phenotype correlation. *Meta Gene*. 2015;7:76‐82. Published 2015 Dec 10. doi:10.1016/j.mgene.2015.12.002

Wahlberg K, Love TM, Pineda D, et al. Maternal polymorphisms in glutathione-related genes are associated with maternal mercury concentrations and early child neurodevelopment in a population with a fish-rich diet. *Environ Int*. 2018;115:142‐149. doi:10.1016/j.envint.2018.03.015

Wang S, Zhang J, Jun F, Bai Z. Glutathione S-transferase pi 1 variant and squamous cell carcinoma susceptibility: a meta-analysis of 52 case-control studies. *BMC Med Genet*. 2019;20(1):22. Published 2019 Jan 21. doi:10.1186/s12881-019-0750-x

Weich N, Ferri C, Moiraghi B, et al. GSTM1 and GSTP1, but not GSTT1 genetic polymorphisms are associated with chronic myeloid leukemia risk and treatment response. *Cancer Epidemiol*. 2016;44:16‐21. doi:10.1016/j.canep.2016.07.008

Yu J, Ahn K, Shin YH, et al. The Interaction Between Prenatal Exposure to Home Renovation and Reactive Oxygen Species Genes in Cord Blood IgE Response is Modified by Maternal Atopy. *Allergy Asthma Immunol Res*. 2016;8(1):41‐48. doi:10.4168/aair.2016.8.1.41

Zhao E, Zhao Y. Lack of association between GSTP1 Ile105Val polymorphism and coronary heart disease risk: a meta-analysis. *Int J Clin Exp Med*. 2015;8(10):18488‐18493.

[www.worldometers.info/coronavirus/countries](http://www.worldometers.info/coronavirus/countries) Assessed July 7, 2020.

<https://data.worldbank.org/indicator/NY.GNP.PCAP.PP.CD> Assessed May 27, 2020.

[www.who.int/countries/en/](http://www.who.int/countries/en/) Assessed May 23, 2020.
